# Role of cancer stem cells in racial disparity in colorectal cancer

**DOI:** 10.1002/cam4.690

**Published:** 2016-03-14

**Authors:** Lulu Farhana, Fadi Antaki, Mohammad R. Anees, Pratima Nangia‐Makker, Stephanie Judd, Timothy Hadden, Edi Levi, Farhan Murshed, Yingjie Yu, Eric Van Buren, Kulsoom Ahmed, Gregory Dyson, Adhip P. N. Majumdar

**Affiliations:** ^1^ Department of Veterans Affairs Medical Center4646 John RDetroitMichigan48201; ^2^ Karmanos Cancer CenterDetroitMichigan48201; ^3^ Department of Internal MedicineWayne State UniversityDetroitMichigan48201; ^4^ Department of Pathology, MedicineWayne State UniversityDetroitMichigan48201

**Keywords:** Adenoma, African‐Americans, cancer stem cells, colorectal cancer, lncRNA, microRNA, miR‐1207‐5p, White Americans

## Abstract

Although African‐Americans (AAs) have a higher incidence of colorectal cancer (CRC) than White people, the underlying biochemical mechanisms for this increase are poorly understood. The current investigation was undertaken to examine whether differences in self‐renewing cancer stem/stem‐like cells (CSCs) in the colonic mucosa, whose stemness is regulated by certain microRNAs (miRs), could partly be responsible for the racial disparity in CRC. The study contains 53 AAs and 47 White people. We found the number of adenomas and the proportion of CD44^+^ CD166^−^ CSC phenotype in the colon to be significantly higher in AAs than White people. MicroRNAs profile in CSC‐enriched colonic mucosal cells, expressed as ratio of high‐risk (≥3 adenomas) to low‐risk (no adenoma) CRC patients revealed an 8‐fold increase in miR‐1207‐5p in AAs, compared to a 1.2‐fold increase of the same in White people. This increase in AA was associated with a marked rise in lncRNA PVT1 (plasmacytoma variant translocation 1), a host gene of miR‐1207‐5p. Forced expression of miR‐1207‐5p in normal human colonic epithelial cells HCoEpiC and CCD841 produced an increase in stemness, as evidenced by morphologically elongated epithelial mesenchymal transition( EMT) phenotype and significant increases in CSC markers (CD44, CD166, and CD133) as well as TGF‐*β*, CTNNB1, MMP2, Slug, Snail, and Vimentin, and reduction in Twist and N‐Cadherin. Our findings suggest that an increase in CSCs, specifically the CD44^+^ CD166^−^ phenotype in the colon could be a predisposing factor for the increased incidence of CRC among AAs. MicroRNA 1207‐5p appears to play a crucial role in regulating stemness in colonic epithelial cells in AAs.

## Introduction

The incidence and mortality of colorectal cancer (CRC), the third most common cancer in the United States and the second leading cause of cancer deaths, is higher among African‐Americans (AAs) than White people 1, 2, 3, 4, 5. African‐Americans tend to be diagnosed with CRC at a younger age and exhibit worse prognoses than their White counterparts 2, 6. It has also been reported that AAs with high‐grade tumor differentiation were three times more likely to die within 5 years after post‐surgery compared to White people with high‐grade tumors 7. However, the underlying reasons for these differences between the two racial groups are poorly understood.

It has been reported that the germ‐line mutation in mismatch repair genes hMLH1 and hMLH2 8, 9 and unique polymorphisms in the p53 tumor suppressor gene are more widespread in colon tumors from AAs than in White people 10, 11, 12. However, since sporadic CRC constitutes about 85% of CRC, higher germ‐line mutations alone cannot fully account for the marked increase in the incidence of CRC, observed among AAs 13, 14. Kang et al. 15 have examined somatic gene mutations in AAs and White people, and found the frequency of mutations in *KRAS, BRAF*, and *PIK3CA* to be higher in AAs than White people suggesting that these increases may in part be responsible for the poor prognosis in CRC among AAs.

The concept that pluripotent cancer stem/stem‐like cells (CSCs) are involved in the development and progression of many malignancies, including CRC is now well accepted and continues to gain credibility as more evidence is uncovered 16, 17, 18. Results from our earlier pilot study demonstrated that the proportion of CSCs, specifically the CD44^+^CD166^−^ CSC phenotype in the colon, shed into the lumen, is greater in patients with adenomatous polyps than those without polyps, and is also higher in AAs with polyps than their White counterparts 19. The primary objective of the current investigation was to elucidate the molecular mechanisms for racial disparity in CRC, focusing mainly on colon CSCs, specifically the CD44^+^CD166^−^ phenotype, and also the role of microRNAs (miRs) in regulating stemness in colon CSCs.

## Methods and Materials

### Cell culture

Human colonic epithelial cells ECoEpiC were purchased from ScienceCell Research Laboratories (Carlsbad, CA) 20 and CCD841, a product of ATCC (American Tissue Culture Collection, Rockville, MD), was provided by Dr. Marc Bissonnette, University of Chicago. Human colon cancer cells HT‐29 and HCT‐116 cells were obtained from ATCC. The cells were maintained in Dulbecco's minimum essential medium (DMEM/F‐12) supplemented with 10% fetal bovine serum (Invitrogen, Grand Island, NY) and 1% gentamycin in humidified incubator at 37°C in an atmosphere of 95% air and 5% carbon dioxide.

### Study subjects and colonoscopy

The study was approved by the Institutional Review Boards and Committees of the John D. Dingell‐Veterans Affairs Medical Center (JDD‐VAMC) and Wayne State University (WSU) School of Medicine. Eligible study subjects were between the ages of 40 and 80 years, scheduled for an outpatient colonoscopy at the JDD‐VAMC. Patients who were excluded from the study were those with active malignant disease, inflammatory bowel disease, recent infection and those with psychiatric or addictive disorder, hemorrhagic diathesis, or on warfarin. Patients were doubly consented, once by the gastroenterologist for the colonoscopy and again by the study coordinator for participation in the study.

All study subjects received standard colonoscopy purgative preparation as per the usual protocol. Briefly patients were asked to stay on a clear liquids diet for 24 h and to take a preparation starting with 15 mg of bisacodyl the morning prior to their colonoscopy. The patients were also instructed to split the dose (4 L) of polyethylene glycol solution (PEG) into a first half (2 L) the evening prior to colonoscopy, and to drink the second half (the remaining 2 L) 5 h prior to the procedure and to finish it 3 h prior to the procedure, regardless of appointment time (morning or afternoon).

### Collection of samples, isolation of colonocytes

During colonoscopy, the retained colonic fluid “effluent” (washings collected during colonoscopy) was aspirated through the working channel of the endoscope and used the same day to isolate colonocytes. Additionally, eight forceps biopsies were taken from macroscopically normal appearing colonic mucosa (<10 cm anal verge). Four biopsies were flash frozen with liquid nitrogen and stored at −80°C, whereas the rest of them were used immediately to isolate colonocytes as described below.

For isolation of colonocytes from colonic effluent, the Somatic Cell sampling and Recovery (SCRC) fecal cell isolation kit (NonInvasive Technologies, Elkridge, MD) was used as described earlier 19. Briefly, approximately 20 mL effluent was diluted with the transporting medium and filtered through a 300‐*μ*m mesh, subsequently passed through 100, 70, and 40 *μ*m filter strainer in sequence. The filtered samples were carefully underlaid with 10 mL warm cushion solution and then centrifuged at 200*g* for 10 min at room temperature. The supernatant was removed and the colonocytes containing interphase was collected, washed 3 times with cold PBS (Phosphate Buffered Saline) (40 mL). The yield was 1–2 × 10^6^ colonocytes per patient. CD44^+^CD166^− ^CSC phenotype was stained, isolated, and quantitated by flow cytometry as described below.

For isolation of mucosal cells from the colon, biopsies were collected in PBS‐containing 10% antibiotic/antimycotic (PBS‐AA) and washed with PBS‐AA five times at 4°C and subsequently incubated overnight in DMEM (Dulbecco's Modified Eagle Medium)/F‐12 medium‐containing 5% antibiotic/antimycotic at 4°C. The tissues were cut into fine pieces using sterile scalpel and then digested with 1.5 mg/mL collagenase I (Sigma‐Aldrich, St Louise, MO) and 20 *μ*g/mL hyaluronidase (Sigma‐Aldrich) under gentle agitation for 2–3 h at 37°C with intermittent mixing. The digested tissues were filtered through 40‐*μ*mol/L mesh, centrifuged at 200g for 5 min and washed with DMEM/F‐12 medium containing 5% antibiotic/antimycotic. The cells were resuspended in stem cells medium and plated in 24‐well low‐adhesion plates (Corning Incorporated, Corning, NY). After 7 days, live cells that primarily represent CSCs were collected, used for further biochemical analysis or subjected to flow cytometric analysis for isolation of CD44^+^CD166^−^ CSC phenotype as stated below.

### Quantitation and sorting of CD44^+^CD166^−^ phenotype by flow cytometry

All reagents and instrumentation used for flow cytometry were from BD Biosciences (San Jose, CA). Colonocytes isolated from effluent or mucosal biopsies were resuspended in 100 *μ*L of 1% FBS in PBS and stained with fluorophore‐conjugated antibodies as follows: with anti‐CD45‐perCP‐Cy5.5 (clone), anti‐CD44‐PECy7 (clone: G44‐26), and anti CD166‐PE (clone) or isotype‐matched mouse IgG1‐PerCP‐Cy5.5, IgG2b‐PE‐Cy7, and PE‐mouse IgG1 K (BD Pharmingen, San Diego, CA). The cells stained with fluorophore‐conjugated antibodies were incubated for 1 h at 4°C, subsequently washed with PBS and resuspended in 0.5 mL PBS. Compo‐Bead plus particles were stained in parallel, in accordance with manufacturer's instructions, to provide compensation controls. CD45^+^ cells were excluded from analysis as described previously 19. Flow cytometry was performed on a FACS Vantage SE SORP and data analyzed with CellQuest.

### RNA isolation and microRNA array

For analysis of microRNA profiling in colon CSCs from AAs and White people, RNA from colon CSCs was extracted from patients without any polyp (controls) and those with no adenomas (low‐risk group) and others with ≥3 adenomas (high‐risk group). The cells were homogenized in Qiazol (Qiagen, Valencia, CA) and RNA was extracted using the miRNeasy kit (Qiagen) according to the manufacturer's protocol.

miRNA expression profiles were performed using the human cancer stem cells array MIHS‐118ZA miScript miRNA SYBR green PCR‐expressing 84 miRNAs. Reverse transcription of RNA for first strand cDNA synthesis and real‐Time (RT) PCR were carried out following the manufacturer's instructions. Data were analyzed using the web‐based software (http://pcrdataanalysis.sabiosciences.com/mirna). Ct values were utilized to assess the relative concentration of miRNA for each sample as described by the manufacturer. ΔCt value for each mature miRNA was calculated using the following formula ΔCt = Ct^miRNA^ − AVE Ct^6internal controls^. A set of controls present for each array was used for data analysis utilizing the ΔΔCt method of relative quantification and fold change was calculated. miRNA signal was normalized using default setting: the threshold raw signal to 0.2 in Applied Biosystems 7500 (Applied Biosystems, Grand Island, NY).

### Quantitative Taqman real time‐PCR

For microRNA (miR) validation, RNA was prepared from CD44^+^CD166^−^ CSCs, HCoEpiC, HT‐29 and HCT116 cells by using miRNeasy kit (Qiagen) according to the manufacturer's protocol. One hundred nanogram of RNA was used to prepare cDNA using TaqMan microRNA reverse transcription kit (Applied Biosystems) using specific microRNA primers. The miR sequence specific primers and probes for miR‐1207, miR‐15a, miR‐31, miR‐10a, miR‐29b, miR‐181a, and miR‐146 endogenous control miR‐26b and PCR used were the same as described previously 21. Data analysis was performed with ΔΔCt values of miRNAs, and each sample was calculated by normalizing with internal control (miR‐26b) and each value represented mean of three replicates.

To evaluate the expression of long noncoding RNA (lncRNA), real‐time SYBR‐green PCR was performed using primers for PVT1 which was purchased from Qiagen Inc. *β*‐actin or U6 served as an internal control.

### mRNA quantitation

Total RNA was prepared from HCoEpiC and CCD841 cell line using TRIzol as recommended by the manufacturer and purified using the Rneasy Mini Kit (Qiagen). For real‐time PCR, cDNA was prepared with the SuperScript III First‐Strand cDNA synthesis system for RT‐PCR (Invitrogen) and analyzed in triplicate using the 2× SYBR Green PCR Master Mix (Applied Biosystems) and the ABI Prism 7500 sequence detection system. PCR consisted of 40 cycles of 95°C for 10 min and then 95°C for 15 sec, 60°C for 60 sec. The primer sequences were used to evaluate the expression of metallopeptidase‐2 (MMP2), Slug, Twist, N‐Cadherin, Vimentin, and CD44, CD166, CD133 as described 22, 23. 2′‐O‐methylated miR‐1207‐5p (an antisense miR to miR‐1207‐5p; 5′‐mC/ZEN/mCmCm mCmUmCmCmCmAmGmCmCmUmCmCmCmUmGmCmC/3ZEN/‐3′) was also used. All oligonucleotide primers as well as the internal control *β*‐actin were synthesized by the Integrated DNA technology Inc. (Coralville, IA). The primer set for each gene is listed below:

CD44 – forward: 5′‐AAGGTGGAGCAAACACAACC‐3′, reverse: 5′‐AACTGCAATGCAAACTGCAAG‐3′; CD166‐ forward: 5′‐TAGCAGGAATGCAACTGTGG‐3′, reverse: 5′‐ CGCAGACATAGTTTCCAGCA‐3′; TGF*β*‐forward: 5′**‐** GTGGAAACCCACAACGAAAT**‐**3′

reverse: 5′‐ CACGTGCTGCTCCACTTTTA**‐**3′; *β*‐catenin Catenin (cadherin‐associated protein) Beta 1 (CTNNB1), forward: 5′‐TGGATGGGCTGCCTCCAGGTGAC‐3′, reverse: 5′‐ACCAGCCCACCCCTCGAGCCC‐3′; MMP2, forward: 5′‐GCTGGCCTAGTGATGATGTTAGGCA‐3′, reverse: 5′‐CCTTGGGGCAGCCATAGAAGGT‐3′; Slug ‐ forward: 5′‐CTACAGCGAACTGGACACACA‐3′, reverse: 5′‐GGAATGGAGCAGCGGTAGT‐3′; Twist‐ forward: 5′‐GTGGCTCACGAGCGGCTCAG‐3′, reverse: 5′‐CTAGGTCTCCGGCCCTGCTG‐3′; N‐Cadherin‐forward: 5′‐CCTGCGCGTGAAGGTTTGCC‐3′, reverse: 5′‐CCAAGCCCCGCACCCACAAT‐3′; Vimentin, forward: 5′‐CGCCCTCGTTCGCCTCTTCT‐3′, reverse: 5′‐GGACATGGCTGCGGAGGGTG‐3′; *β*‐actin‐forward: 5′‐CCCAGCACAATGAAGAATCAA‐3′, reverse: 5′‐ACATCTGCTGGAAGGTGGTGGAC‐3′.

### Statistical analysis

All statistical analyses were performed using VassarStat web software (Richard Lowry, Poughkeepsie, NY). One‐way analysis of variance (ANOVA) was performed to detect any differences between the two races for different parameters. If the result of the ANOVA is significant, pairwise comparisons between the groups were made by a post hoc test (Turkey's HSD procedure). A value of *P < 0.05* was taken as the level of significance.

## Results

### Baseline characteristics of study subjects

A total of 110 patients were included in this study. Average age of the patients was 63 years, and 93% of them were males (Table 1). The average body mass index (BMI) was 30.0. However, White people exhibited a slightly higher BMI than AAs (Table 1). The number of adenomas was found to be significantly higher (48%; *P < 0*.04) in AAs than White people (Table 1).

**Table 1:** Baseline characteristics of the subjects

Characteristics	AA	White people	*P*‐value
Number of patient (*n*)	63	56	
Age (year)	63.4 ± 6.7	63.1 ± 8.1	
Gender (male)	93%	93%	
Body mass index (mean)	28.7 ± 5.9	31.2 ± 6.9	0.03
Patients with adenomas (*n*)	42	36	
Adenomas per patient	3.7 ± 2.9	2.5 ± 2.0	0.04

AA, African American.

Values are expressed as mean ± SD.

The outcome of variable of adenoma status was determined and those classified as “high‐risk” (HR) had advanced adenomatous polyps with villous or mixed tubulovillous features with high‐grade dysplasia (HGD) or their polyp size being ≥1.0 cm size. This group included 19 patients; AA (*n* = 10) and White people (*n* = 9). The number of patients with ≥3 tubular adenomas, also classified as HR was 17, and those who had no tubular adenomatous polyps were termed as “low‐risk” (LR) group. In majority (73.7%) of patients, adenomas were located in the proximal colon, and 26.3% were found in the distal colon and 7% were in the rectum. In both AAs and White people, the number of adenomas was found to be significantly higher in proximal than distal colon (AAs; mean ± SD; proximal = 2.81 ± 0.35 vs. distal 1.70 ± 0.31; *P* < 0.045) and (White people; mean ± SD; proximal 2.63 ± 0.33 vs. distal 1.43 ± 0.15; *P* < 0.0064). The number of adenomas in the rectum was slightly higher in AAs (11%) than White people (3%).

### CD44^+^CD166^−^ CSC phenotype is more prevalent in the colon of AAs than Whites

**Figure 1:**
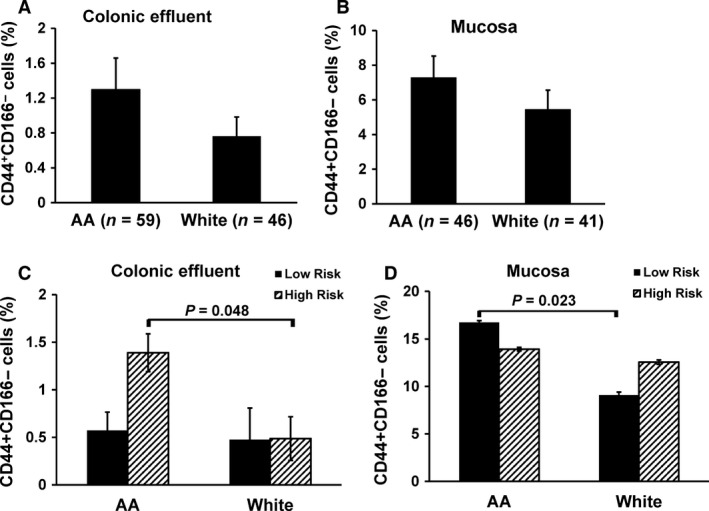
Flow‐cytometric analysis of colonocytes showing changes in the proportion of CD44^+^ CD166^−^ cancer stem cells phenotype (A & B) in colonic effluent and mucosa and (C & D) in the colon of “high‐risk” (HR; ≥3 adenomas) and “low risk” (LR; no adenoma) for colorectal cancer in African‐Americans (AAs) and White people.

Although the underlying molecular mechanisms for increased incidence of CRC in AAs are not fully understood, we suggested a role for CSCs in regulating this process 19. In support of this postulation, we reported that the proportion of CD44^+^CD166^−^ CSC phenotype in the colon, which is higher in patients with tubular adenomas than those without adenomas, is also higher in the colon of AAs than White people 19. Our current data further demonstrate that the number of adenomas in AAs, which is significantly higher (48%) than White people (Table 1), is also associated with 50–80% higher CD44^+^CD166^−^ CSC phenotype in the colonic effluent and colonic mucosa from AAs than their White counterparts (Fig.**** 1A and B). When the patients were stratified as HR and LR for CRC, we found the proportion of CD44^+^CD166^−^ phenotype from the colonic effluent of HR AAs to be 170% higher than their White counterpart (Fig.**** 1C). No change in CD44^+^CD166^−^ from colonic effluent was observed between the two races in LR group (Fig.**** 1C). In contrast, the proportion of CD44^+^CD166^−^ phenotype, isolated from colonic mucosal cells of LR AAs was found to be significantly higher (84%) than their White counterparts (Fig.**** 1D).

### microRNAs profile in the colon of AAs and White people

The microRNAs (miRs) are a class of 18–24 nucleotide long endogenous noncoding RNAs that control gene expression through binding to the seed sequence at the 3′‐UTR of target mRNAs, resulting in translational repression or mRNA degradation 24. microRNAs can perfectly or imperfectly base pair with multiple targets, allowing it to potentially regulate the translation of several miRs. It has been predicted that over 30% of the human protein coding genes are also posttranscriptionally regulated by this mechanisms 25, 26, 27, 28. miRs are also known to regulate various properties of CSCs 24, 29. To determine whether miRs play a role in regulating CSCs in the colon of AAs and White people, we examined the miR profile in colonic mucosal CSCs from AAs and White people, stratified with and without adenomas, using human cancer stem cell microarray.

Microarray analysis revealed at least 18 miRs to be upregulated and 3 miRs downregulated in AAs and White people with adenomas versus without adenoma (data not shown). We selected seven miRs based on variation in expression levels between two races. We found five miRs to be upregulated (miR‐1207‐5p, miR‐31‐5p, miR‐181a, miR‐146a‐5p, and miR‐15a‐5p) and 2 miRs downregulated (miR‐29b‐3p, miR‐10a) in AA (Table 2)**.** While, 4 miRs (miR‐31, miR‐29b, miR‐10a, and miR‐15a‐5p) were upregulated, three miRs showed either little change in level of expression (miR‐1207‐5p and miR‐181a) or were downregulated (miR‐146b‐5p) in White people (Table 2). However, when miR levels for each were calculated as the ratio of HR to LR for each racial group, we noted over 8‐fold increase in miR‐1207‐5p in AAs, whereas in White people the increase was found to be only about 1.2‐fold in colon CSCs (Table 2). These changes in miRs levels between the two racial groups prompted us to examine the role of miR‐1207‐5p in regulating racial disparity in CRC.

**Table 2:** miRNAs profile in colonic mucosal cells enriched in cancer stem/stem‐like cells from normal colonic mucosa of African‐Americans and White people

miRNA	African‐American (AA)			White people		
	Log2 value		Fold	Log2 value		Fold
	HR(Adenoma)	LR(No adenoma)		HR(Adenoma)	LR(No adenoma)	
miR‐1207‐5p	0.81	0.094	8.63	0.36	0.296	1.21
miR‐31‐5p	0.055	0.012	4.58	0.04	0.008	4.4
miR‐181a	0.18	0.039	4.69	0.13	0.12	1.11
miR‐29b‐3p	0.087	0.221	0.39	0.30	0.12	2.53
miR‐146b‐5p	0.35	0.034	10.3	0.18	0.37	0.49
miR‐10a	0.006	0.018	0.33	0.53	0.018	2.88
miR‐15a‐5p	0.23	0.035	6.57	0.44	0.133	3.31

HR, high risk; LR, low risk.

Racial differences in miRs were validated by TaqMan qRT‐PCR in CD44^+^CD166^−^ CSC phenotype, isolated from AAs and White people with and without adenomas. A pattern was slightly different (such as miR‐146b) in CD44^+^CD166^−^ CSC phenotype than that was observed in mucosal CSC (Table 2). A similar pattern was observed for miR‐1207‐5p in CD44^+^CD166^−^ CSC phenotype, whereas for other 6 miRs, the same ratio did not reveal a marked change between the two racial groups (Tables 2 and 3).

**Table 3:** Quantitative real‐time PCR showing relative expression of miRNAs in CD44^+^ CD166^−^ CSC phenotype from colonic mucosa of African‐Americans and White people

miRNA	African‐American (AA)				White people			
	Log2 value		Fold	*P*‐ value	Log2 value		Fold	*P*‐ value
	HR(Adenoma)	LR(No adenoma)			HR(Adenoma)	LR(No adenoma)		
miR‐1207‐5p	0.26	0.18	1.4	*	0.24	0.25	0.93	—
miR‐31‐5p	0.25	0.19	1.3	*	0.23	0.16	1.4	*
miR‐181a	0.01	0.00	1.8	**	0.01	0.005	1.6	*
miR‐29b‐3p	0.0009	0.003	0.25	**	ND			
miR‐146b‐5p	3.10	2.64	1.17	—	3.25	2.78	1.16	—
miR‐10a	0.15	0.09	1.57	*	0.12	0.06	2.02	**
miR‐15a‐5p	0.003	0.00	0.17	***	0.004	0.007	1.5	**

HR, high risk; LR, low risk.

*P<0.05; **P<0.01; ***P < 0.001.

**Figure 2:**
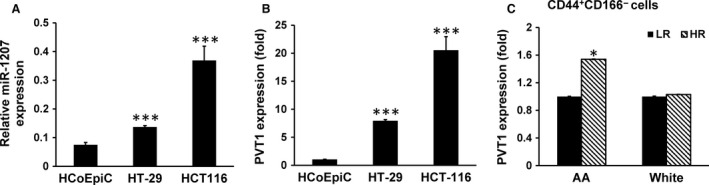
Quantitative real‐time PCR showing changes in relative levels of microRNA (miR)‐1205‐5p and lncRNA PVT1 in normal colonic epithelial cells (HCoEpiC) and colon cancer HT‐29 and HCT‐116 cells (A and B). Increased PVT1 expression in mucosal CD44^+^ CD166^−^ cancer stem cells phenotype of African‐Americans (C). Results are expressed as mean ± standard deviation (SD) of three separate experiments. *P < 0.05 and ***P < 0.00.

Prior studies have shown miR‐1207‐5p to be upregulated in colon tumors 30. Given the magnitude of increase in miR‐1207‐5p in the colonic mucosa of HR in AAs than their White people counterparts prompted us to further examine whether miR‐1207‐5p could be involved in regulating CSCs in AAs. Initial studies were carried out to determine whether miR‐1207‐5p is dysregulated in carcinogenesis. Indeed, we observed that the expression of miR‐1207‐5p was markedly higher in colon cancer HT‐29 and HCT‐116 cells, compared to normal human colonic epithelial cells HCoEpic (Fig. 2A). Likewise, the expression of long noncoding RNA (lncRNA) PVT1, the host gene of miR‐1207‐5p was found to be approximately 9‐fold and 20‐fold higher in colon cancer HT‐29 and HT‐116 cells, respectively, compared to normal human colonic epithelial cells, HCoEpiC (Fig.**** 2B). In line with these observations, we also found that the expression of PVT1 in CD44^+^CD166^−^ CSC phenotype from HR AAs to be significantly higher (~70%) than their White counterparts (Fig.**** 2C). No apparent difference in PVT1 expression was observed in “low‐risk” patients between the two racial groups (Fig.**** 2C).

**Figure 3:**
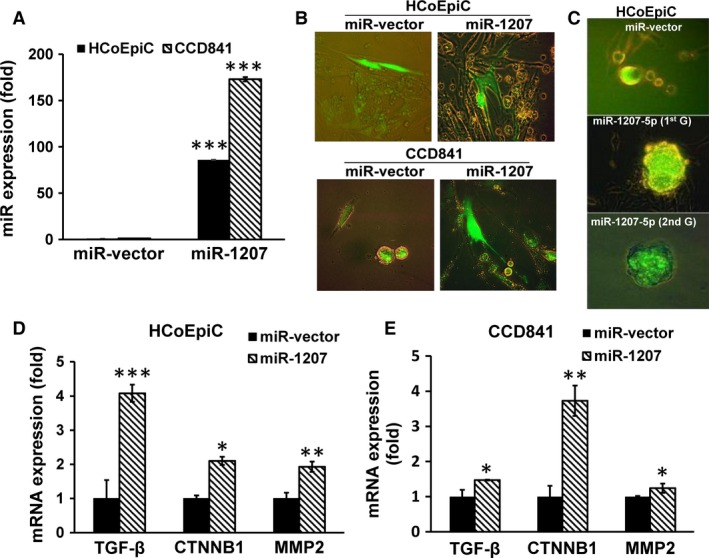
Forced expression of pre‐microRNA (miR)‐1207‐5p in HCoEpiC or CCD841 cells that caused a marked increase in miR‐1207‐5p levels (A), produced changes in morphology to epithelial mesenchymal transition phenotype as shown in the photomicrograph (B), formed spheroids following incubation for 14 days in medium containing serum [first generation; 1st G], which were disintegrated and the cells subsequently incubated in serum‐free stem‐cell medium for another 14 days [second generation; 2nd G] (C), accompanied by significant increases in transforming growth factor‐*β*‐receptor, Catenin (cadherin‐associated protein)‐*β*‐1 and MMP2 expression (D and E). Photomicrographs were taken under 400× magnification using Olympus fluorescence microscope with digital camera and DP2‐BSW software. Where applicable, data are presented as mean ± SD of three separate experiments. *P < 0.05, **P < 0.01 and ***P < 0.001.

**Figure 4:**
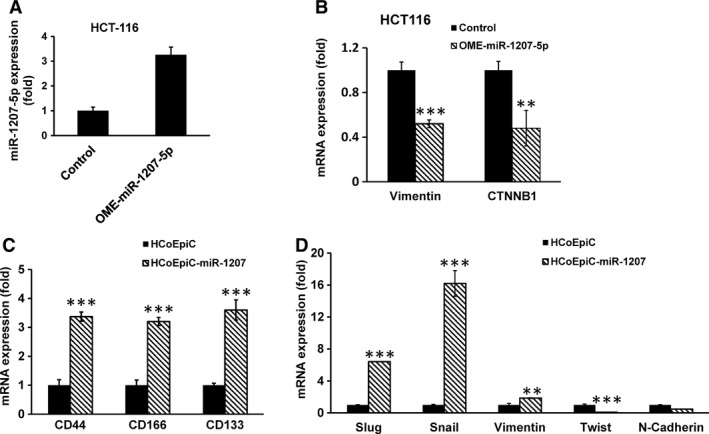
Although transfection of O'methylated microRNA (miR)1207‐5p (OME‐miR‐1207‐5p, a functional inhibitor of miR1207‐5p) in HCT‐116 cells caused a marked increase in the expression of miR1207‐5p (A), it resulted in a marked reduction in Vimentin and Catenin (cadherin‐associated protein)‐*β*‐1 expression, compared to the control (B). Forced expression of pre‐miR‐1207‐5p in HCoEpiC cells significantly increased the expression of colon cancer stem cell markers CD44, CD166, and CD133 as well as the levels of Slug, Snail and Vimentin, but caused a decrease in Twist and N‐ Cadherin (C and D). Data represented mean ± SD of three separate experiments. **P < 0.0.01 and ***P < 0.001.

To further determine the role of miR‐1207‐5p in regulating stemness in colonic epithelial cells, miR‐1207‐5p was forced‐expressed in normal human colonic epithelial cells, HCoEpiC and CCD841. Forced expression of miR‐1207‐5p in HCoEpiC and CCD841 cells resulted in approximately 85‐fold and 170‐fold higher expression, respectively, than the corresponding miR‐vector‐transfected controls (Fig. 3A). Overexpression of miR‐1207‐5p in HCoEpiC and CCD841 cells resulted in changes in morphology of these normal human colonic epithelial cells to more elongated epithelial mesenchymal transition phenotype (EMT) (Fig. 3B). In addition, we observed that forced expression of miR‐1207‐5p in HCoEpiC cells caused spheroids to form following 14 days incubation in serum containing medium. We also observed that when the initial miR‐1207‐induced spheroids (first generation) were disintegrated and the cells incubated again in serum‐free stem‐cell media, they readily formed spheroids (second generation) (Fig. 3C), indicating self‐renewal ability of cancer stem cells. These changes were associated with marked increases in TGF‐*β*, CTNNB1, and MMP2 (Fig. 3D and E), each of which is known to be upregulated in CSCs 31, 32, 33. In contrast, transfection of 2′‐O‐methylated‐miR‐1207‐5p (functional inhibitor of miR‐1207‐5p) in HCoEpiC caused a significant inhibition (~50%) in CTNNB1 and Vimentin, compared to the miR control (Fig. 4B). It should be noted that functional inhibition of miR‐1207 by OME‐miR‐1207‐5p resulted in a marked increase in miR‐1207 expression in HCT‐116 cells (Fig. 4A), a phenomenon that has been noted by others 34, 21.

The change in EMT‐like morphology of HCoEpiC and CCD841 cells following forced expression of miR‐1207‐5p was also associated with a marked rise in the expression of colon CSC markers CD44, CD166, and CD133 (Fig. 4C). In addition, levels of Slug, Snail, and Vimentin were increased, and Twist and N‐Cadherin were decreased in HCoEpiC cells following overexpression of miR‐1207‐5p (Fig. 4D). Taken together, these observations suggest a role for miR‐1207‐5p in inducing stemness in colonic epithelial cells.

## Discussion

Although the incidence of colorectal cancer (CRC) has been found to be higher in AAs than in White people, the underlying mechanisms for this racial disparity are poorly understood. CSCs with self‐renewing ability are thought to be a predisposing factor for the development of CRC 35, 36, 37. It is becoming increasingly evident that many epithelial cancers, including CRC arise from a small subpopulation of CSCs through oncogenic transformations 38. In support of this postulation, we have demonstrated the presence of CSCs in the colon of humans, as evidenced by the expression of CD44, CD166, and ESA not only in premalignant adenomatous polyps, but also in normal appearing colonic mucosa, where expression of several CSC markers was found to increase with advancing age indicating increased risk in developing CRC with aging 35, 39, 40, 41. We also reported that expression of CSC markers in normal appearing colonic mucosa is about twofold higher in subjects with 3–4 polyps than those with 1–2 polyps 39. The expression of CSC markers tends to be higher in other premalignant conditions in the gastrointestinal tract such as chronic gastritis and *Helicobacter pylori*‐infected gastric mucosa as well 42, 43. Taken together, these observations supported the contention that CSCs could be a predisposing factor for the development of gastrointestinal cancers, including CRC.

In view of this, we previously examined the differences in CSCs in the colon of AAs and White people and found the proportion of CSCs, specifically the CD44^+^CD166^−^ phenotype in the colonic effluent of AAs with adenomas to be higher in AAs than their White counterparts 19. Our current observation that the proportion of CD44^+^CD166^−^ in the colonic effluent and in freshly isolated colonic mucosal cells is markedly higher in AAs with adenomas than their Whites counterparts further strengthens our contention that CSCs, specifically the CD44^+^CD166^−^ phenotype could one of the underlying factors for the increased incidence of CRC in AAs. Further support for this contention comes from the observation that whereas the colonic effluent from AAs with ≥3 adenomas (high‐risk subjects) contains significantly higher proportion of CD44^+^CD166^−^ than their “low‐risk” counterparts (subjects without adenoma), no such difference could be noted among White people. These differences could not be attributed to age or BMI, each of which was similar between the two racial groups.

Although the precise mechanism(s) by which stemness is induced in colonic mucosal cells is not fully understood, it is becoming increasingly evident that miRNAs (miRs) are critically involved in regulating this process 24. Earlier, we reported that miR‐21, an oncomir, to induce stemness in colon cancer cells by downregulating transforming growth factor‐β‐receptor 44. More recently, we reported miR‐21 and the tumor suppressor miR‐145, each of which regulate each other, are also involved in modulating stemness in colon cancer cells 29. Our current data also suggest a role for miRs in modulating stemness in colonic mucosal cells of AAs with adenomas. This inference comes from analysis of miR profile of colonic mucosal cells enriched in CSCs which revealed a marked upregulation of miR‐1207‐5p not only in CSC‐enriched colonic mucosal cells but also in CD44^+^CD166^−^ phenotype from AAs with adenomas. We demonstrated that miR‐1207‐5p expression is upregulated significantly in HCT‐116 and HT‐29 colon cancer cell lines compared to normal colonic epithelial cells, HCoEpiC and CCD841.

It has been reported that miR‐1207‐5p is upregulated in CRC tissues and a wide variety of cancer tissues and cell lines 30, 45, 46. Our observation that forced expression of miR‐1207‐5p in human colonic epithelial cells, HCoEpiC and CCD841 alters their morphology to a more elongated EMT phenotype–a marker for invasion and metastases and causes an increased expression of a number colon CSC markers which among others include CD44, CD166, and CD133 as well as Slug, Snail, and Vimentin further suggests an involvement of this miR in induction of stemnness in colonic epithelial cells. Tran et al. 47 have reported that while Snail1 is uniquely required for EMT initiation, Twist1 is needed to maintain late EMT in both nontumorigenic and tumorigenic epithelial cells. Indeed, our results show a marked increase in Snail expression 48 h following transfection of miR‐1207 in HCoEpiC cells, while Twist and N‐Cadherin decreased during the early phase of EMT development. However, the underlying mechanisms for reduction in Twist and N‐Cadherin remain to be determined.

Further support for miR‐1207‐5p induction of stemness in colonic epithelial cells comes from the observation that the normal human colonic epithelial HCoEpiC cells readily form spheroids‐like structures following forced expression of miR‐1207‐5p. Moreover, the fact that second generation spheroids could also be readily formed in miR‐1207‐5p overexpressing cells suggests that this miR induces self‐renewal, one of the primary properties of CSCs. Taken together, the results suggest that the increased proportion of cancer stem/stem‐like cells in the colon of AAs with adenomas over their White counterparts could partly be result of induction of stemness of mucosal cells by miR‐1207‐5p.

Although the underlying molecular mechanisms for upregulation of miR‐1207‐5p in the colonic mucosa of AAs is unknown, our observation of an increased PVT1, a lncRNA, and p53‐inducible target gene downstream of well‐known cancer risk locus MYC, 48, 49, 50, 51 suggests a role of this lncRNA in regulating this process. Indeed, PVT1 expression has been linked to tumor cells and knockdown of PVT1 from MYC‐driven colon cancer cells has been shown to reduce tumorigenic potency 52. Our observation of significantly elevated levels of PVT1 transcript in the colon of high‐risk AAs, but not in White patients, further supports our contention that PVT1 may play an important role in regulating the miR‐1207‐5p modulation of stemness in colonic mucosal cells in AAs.

In conclusion, our current data show that AAs exhibit a significantly higher number of adenomas than their White counterparts, accompanied a marked increase in the proportion of CSCs in the colon, specifically CD44^+^CD166^−^ phenotype. These increases are associated with a substantial rise in miR‐1207‐5p in patients who are considered to be at higher risk for CRC and an increase in PVT1, the host gene of miR‐1207‐5p. Moreover, our observation that forced expression of miR‐1207‐5p in normal human epithelial cells enhances stemness suggests that miR‐1207‐5p plays a pivotal role in regulating stemness in colonic mucosal cells and that an increase in miR‐1207‐5p in colon CSCs of AAs could be one of the underlying factors for the increased incidence of CRC observed in AAs.

## Conflict of Interest

None declared.
